# Complete genome sequence of a penicillin-resistant *Fusobacterium necrophorum* subsp. *funduliforme isolate* from a tonsillitis patient

**DOI:** 10.1128/mra.01233-24

**Published:** 2024-12-20

**Authors:** Bibek G C, Anders Jensen, Chenggang Wu

**Affiliations:** 1 Department of Microbiology & Molecular Genetics, The University of Texas Health Science Center, Houston, Texas, USA; 2 Department of Clinical Microbiology, Sygehus Lillebælt, Vejle, Denmark; Wellesley College Department of Biological Sciences, Wellesley, Massachusetts, USA

**Keywords:** *Fusobacterium necrophorum*, penicillin-resistant, tonsillitis, bacterial type 4 secretion system, leukotoxin

## Abstract

We report the complete genome sequence of a penicillin-resistant *Fusobacterium necrophorum* subsp. *funduliforme* isolate, AJ79, from a tonsillitis patient. The AJ79 genome consists of a chromosome (2,440,359 bp) and plasmid (9,887 bp), providing insights into the genetic basis of penicillin resistance in *F. necrophorum* and its implications for treating tonsillitis.

## ANNOUNCEMENT

*Fusobacterium necrophorum* is a gram-negative, non-motile anaerobe with two subspecies: *F. necrophorum* subsp. *necrophorum* (FNN) and *F. necrophorum* subsp. *funduliforme* (FNF) (1). FNN is linked chiefly to animal infections, while FNF is mainly isolated from humans (2). Phylogenetic analysis identified two FNF clades, I and III (3). FNN cells typically form filaments, and FNF appears as short rods or coccoid bodies. The main virulence factor, leukotoxin (lktA), is common to both subspecies (3). Penicillin resistance, though rare (6%), may be underreported due to misidentification of *Fusobacterium* spp. (4, 5). No endogenous plasmids have been previously reported in *F. necrophorum* (6).

**Fig 1:**
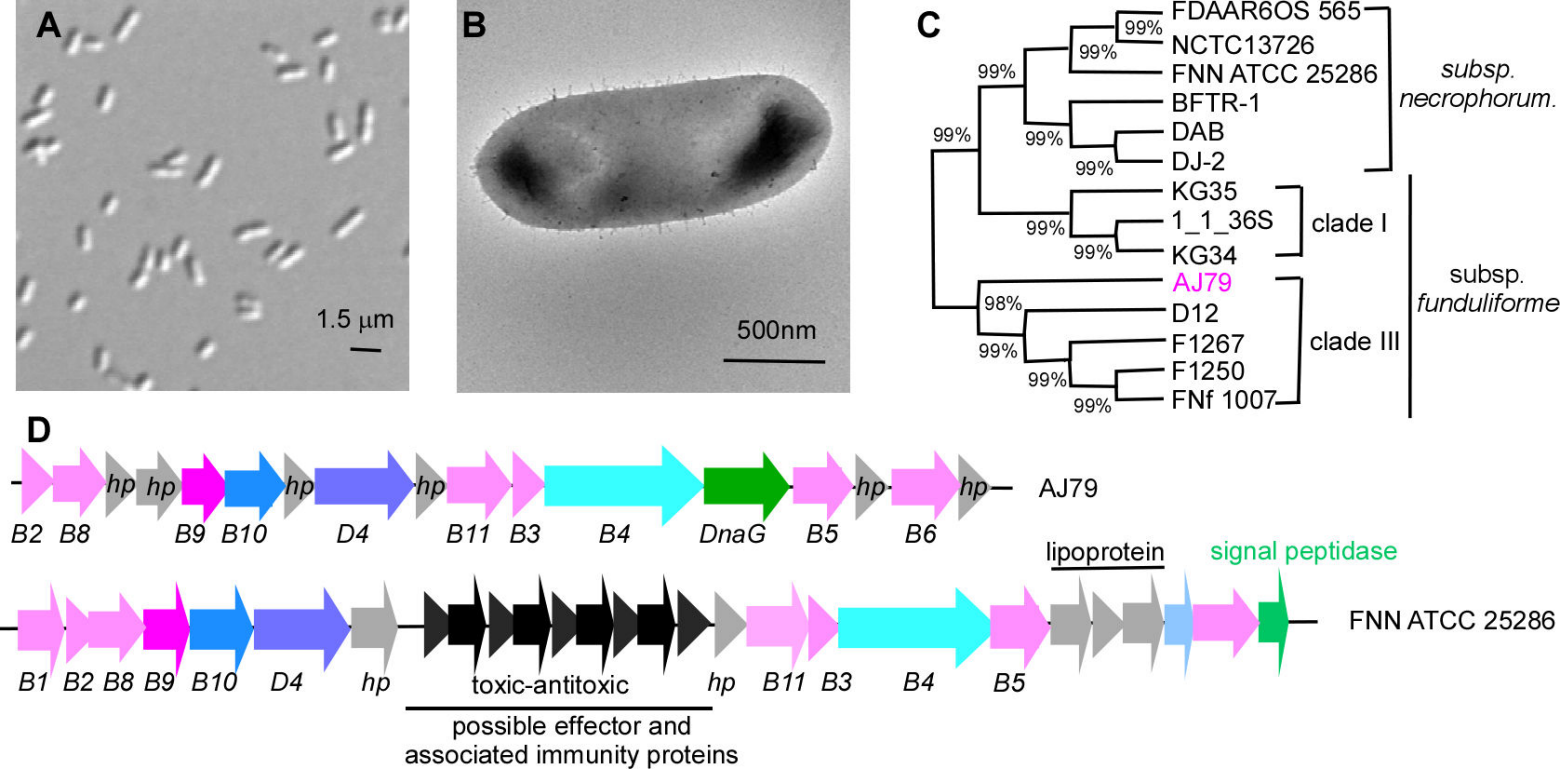
Morphological and phylogenetic analysis of the *Fusobacterium necrophorum subsp. funduliforme* isolate AJ79. (**A**) Light microscopy image of the AJ79 strain shows its short-rod morphology. (**B**) Electron microscopy image revealing small surface protrusions on the cell surface of AJ79. (**C**) Phylogenetic tree analysis of the *lktA* gene, placing AJ79 within clade III of *Fusobacterium necrophorum subsp. funduliforme*. The phylogenetic tree was constructed using the maximum likelihood method in MEGA-X software with 1,000 bootstrap replications, with the bootstrap value shown at each node. (**D**) Gene arrangement of the Type 4 Secretion System (T4SS) operon in AJ79 compared to the FNN strain ATCC 25286.

The AJ79 strain was isolated on 5% blood agar (SSI, Denmark) from a throat swab of a tonsillitis patient in Denmark, with incubation at 37°C for 48 hours under anaerobic conditions. Penicillin susceptibility of strain AJ79 was evaluated using the disc diffusion method (7), and the zone for penicillin was 0 mM. In addition, the positive β-lactamase production was detected using the nitrocefin method. This strain exhibits short-rod morphology (Fig. 1A). Electron microscopy showed small surface protrusions (Fig. 1B). Sequence analysis of the *lktA* genes placed AJ79 in clade III of FNF (Fig. 1C). For whole-genome sequencing, strain AJ79 was revived from a −80°C glycerol stock by streaking onto TSPC agar plates and incubating 10 mL culture anaerobically (85% N₂, 10% CO₂, and 5% H₂) at 37°C. The cells were collected, and genomic DNA was extracted using the Quick-DNA HMW MagBead Kit (Zymo Research; Catalog no. D6060). DNA concentration was measured using a Qubit 4 fluorometer (Thermo Fisher Scientific; Catalog no. Q33327). CD Genomics (Shirley, NY) performed Illumina short-read sequencing and Oxford Nanopore Technologies (ONT) long-read sequencing with *de novo* assembly. Default parameters were used except where otherwise noted.

Short-read libraries were prepared with the Illumina DNA Prep Kit, and paired-end sequencing was performed on an Illumina NovaSeq 6000 platform. For ONT sequencing, libraries were prepared using the Native Barcoding Kit 24 V14 (SQK-NBD114.24) according to the manufacturer’s specifications (no DNA size selection/shearing). Sequencing was performed on a Nanopore R10.4.1 flow cell, and base calling was performed using Dorado on the ONT GridION device. Illumina paired-end reads (2 × 150 bp), and ONT long reads were provided as FASTQ files (Illumina: 5,145,694 reads, 692,937,058 bases, 281× coverage; ONT: 5,706,948 reads, 763,650,186 bases, 311× coverage, with an N50 read length of 2,440.352 kb). Quality control and adapter trimming were performed using bcl2fastq v2.20.0.445 (8) and Porechop v0.2.3_seqan.2.1.1 (9) for Illumina and ONT sequencing, respectively. Hybrid assembly was conducted using Unicycler v0.4.8 (10), integrating both Illumina and ONT reads. The assembly statistics were recorded using QUAST v5.0.2 (11) and annotated using the Prokaryotic Genome Annotation Pipeline (PGAP) v5.2 (12). Interestingly, its genome encodes a complete operon encoding the bacterial Type 4 secretion system (T4SS), absent in other sequenced FNF strains. While the T4SS is commonly found in FNN strains (2), the gene arrangement within the T4SS operon of AJ79 differs from that of ATCC 25286 in FNN (Fig. 1D) (13).

## Data Availability

The whole-genome sequence of Fusobacterium necrophorum strain AJ79 and the endogenous plasmid pAJ01 have been deposited in GenBank under the accession numbers CP169296 and CP169297. The raw sequence reads for Illumina and ONT are accessible under Sequence Read Archive (SRA) accession no. PRJNA1157909.
